# Effectiveness of virtual simulation and jaw model for undergraduate periodontal teaching

**DOI:** 10.1186/s12909-021-03064-1

**Published:** 2021-12-14

**Authors:** Jie Zhang, Jiawei Xing, Min Zheng, Jie Sheng, Kailiang Zhang, Baoping Zhang

**Affiliations:** 1 School of Stomatology Lanzhou University, Lanzhou, 730000 China; 2 Hospital of Stomatology, Lanzhou University, Lanzhou, 730000 China; 3 Gansu Province Key Lab of Maxillofacial Reconstruction and Intelligent Manufacturing, Lanzhou, 730000 China; 4 Gansu Province Clinical Research Center for Oral Diseases, Lanzhou, 730000 China

**Keywords:** Dental education, Virtual reality, Periodontology

## Abstract

**Background:**

The current study explored the effect of virtual simulation and jaw model on development of preclinical periodontal skills in undergraduate students. The study also sought to explore effectiveness of VR in periodontal preclinical training and determine adequate performance mode in basic periodontal education to improve future preclinical training strategies.

**Methods:**

Sixty volunteer sophomores and juniors from the stomatology department in Lanzhou university were enrolled to the current study. Participants were randomly assigned into four groups (each group, *n* = 15) including the traditional jaw model group (Group J) which was the control group, virtual reality group (Group V), virtual-jaw group (Group V-J), and jaw-virtual group (Group J-V). Participants received training on uniform basic periodontal knowledge before completing the first theoretical assessment. Participants further underwent a total 8 h of operation training and completed a second theoretical assessment. Performance of participants was evaluated using the supragingival scaling processes, and clinical operation scores were graded by a blinded professional using an established standard scoring system.

**Results:**

The findings showed no significant difference in the first theoretical outcomes between the four groups (*P* > 0.05). The scores of the second theoretical assessment were significantly improved for the V-J and J-V groups (60.00 ± 4.47, 58.33 ± 4.35) compared with the scores of the first theoretical exam (49.67 ± 4.81, 48.00 ± 4.93, *P* < 0.05). The operation process scores of students in Group V-J and J-V (72.00 ± 5.92; 70.00 ± 3.05) were significantly higher compared with the scores in the other two groups (V: 61.67 ± 7.85; J: 60.67 ± 2.58). The scaling process performance of students in Group V-J and J-V (53.00 ± 3.05; 63.40 ± 4.39) was improved compared with that of students in the other two groups (V: 41.90 ± 5.23; J: 47.40 ± 4.31).

**Conclusion:**

The findings show that combination of virtual reality and jaw model during periodontal preclinical training increases students’ grades and improves acquiring of professional skills. Findings from the current study indicate that the jaw model should be applied prior to virtual reality to ensure high efficacy.

**Supplementary Information:**

The online version contains supplementary material available at 10.1186/s12909-021-03064-1.

## Background

Virtual reality (VR) mimics the real world, and it is widely used in various fields of preclinical training of medical students, such as dental education, surgical skill training, treatment and diagnosis of disease [1]. Notably, this novel pattern is playing an increasingly key role in preclinical training of dentist, and also adequate preclinical training is essential for successful career development of dentists. Previous studies report increasing awareness of the importance of improved teaching skills during undergraduate education [2, 3]. But traditional preclinical training in stomatology is mainly conducted using the simulation jaw model, and the pattern is unitary, limiting its application owing to the complex clinical manifestations of the disease. In addition, students undergoing the training do not receive realistic feedback timely, resulting in low efficiencies and poor training effects [4].

Advances in accessibility of computer-assisted VR technology in dental research has resulted in development of various dental virtual simulation systems for use in oral operation training and preclinical training in some colleges [5–7]. A previous study reported manual agility using tactile VR technology in preclinical dental education and reported that the VR simulator plays a significant role in identifying students who experience learning challenges during the preclinical phase of dental training [8]. Another study explored a training simulator for inferior alveolar nerve blockage and reported that it was suitable for training on needle appropriate positioning, insertion depth, and resistance sensitivity of virtual tissues [9]. It is no difficult to find that virtual simulation teaching mainly focused on simulated maxillofacial surgical [10], local anaesthesia [11], and dental pulp surgical trainings [12]. However, a few studies explored the virtual simulation training application in periodontal teaching [13–15], so it is of importance to establish an effective model of preclinical periodontal teaching.

Periodontitis is an inflammatory disease caused by various factors mainly of bacterial source [16]. It is characterized by a high prevalence affecting approximately 5.4 billion people worldwide [17]. If left untreated, periodontitis can cause damage to dental support tissue, tooth loss and systemic effects [18]. Chronic kidney disease, endothelial dysfunction [19, 20], and coronary heart disease [21] are associated with periodontitis, so effective and timely treatment is critical. Routine practice for a soft tissue examination and periodontal intervention treatment in clinical settings comprises periodontal probing, supragingival and subgingival scaling, and root planing, which are essential clinical skills in training of undergraduates. It is widely known that the traditional jaw model training is a conventional approach for undergraduate education of periodontology. Actually, students often need to spend a lot of time to achieve satisfactory results [22]. Therefore, how to obtain an efficient training model is worth exploring.

This study applies virtual simulation technology to undergraduate preclinical periodontal training, and combines it with traditional jaw training. To compare the influence of different training methods and the order of different training methods on training effect, the findings will provide a new mentality for periodontal preclinical training to further improve clinical skills.

## Methods

The current study was approved by Ethics Committee of the School of Stomatology Lanzhou University (No. LZUKQ-2019-25). All students enrolled in the study provided written informed consent. The duration of the training was 14 h. All experimental protocols involving human subjects were conducted in accordance with the Declaration of Helsinki (2013).

### Participants

A total of 60 volunteers (40 females/20 males) were randomly enrolled to the study and 4 professional clinical doctors (more than 5 years clinical experiences) carried out training of participants. The volunteers were second- and third-year undergraduate students pursuing Stomatology at Lanzhou University. All participants presented with complete dentition, healthy periodontal tissue, no calculus (especially incisors and first molars), no evident malocclusion or any other systemic diseases. Age of participants ranged from 19 to 20 years and analysis showed no significant difference in age. Participants were assigned to the following four groups (each group *n* = 15,10 females/5 males): (1) Jaw model group (Group J) which was the control group, (2) Virtual reality group (Group V), (3) virtual-jaw (Group V-J, virtual simulation training before using the jaw model) and (4) Jaw-Virtual group (Group J-V, used the jaw model before virtual simulation training). Notably, students did not undergo periodontology prior to the study, and the same starting point was used for all subjects.

### Study procedure

#### Theoretical knowledge teaching

**Fig. 1:**
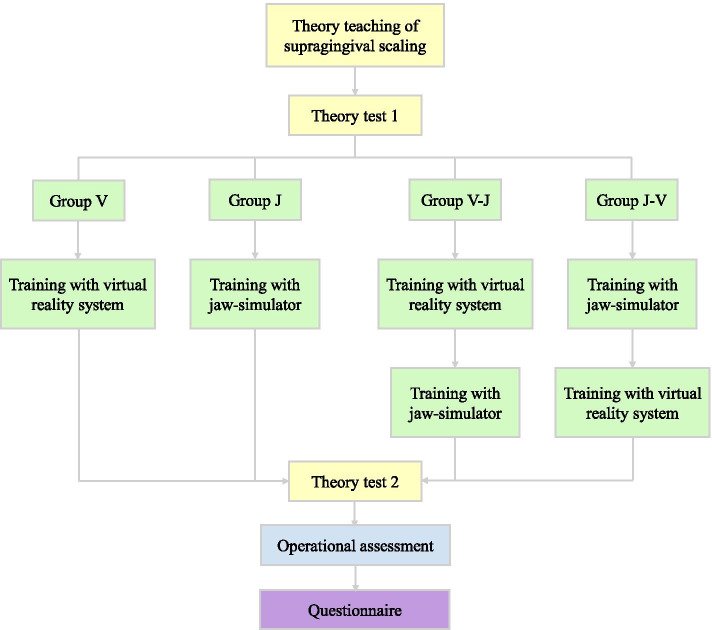
A flow diagram showing use of the virtual reality system and the jaw simulation model for supragingival scaling

**Fig. 2:**
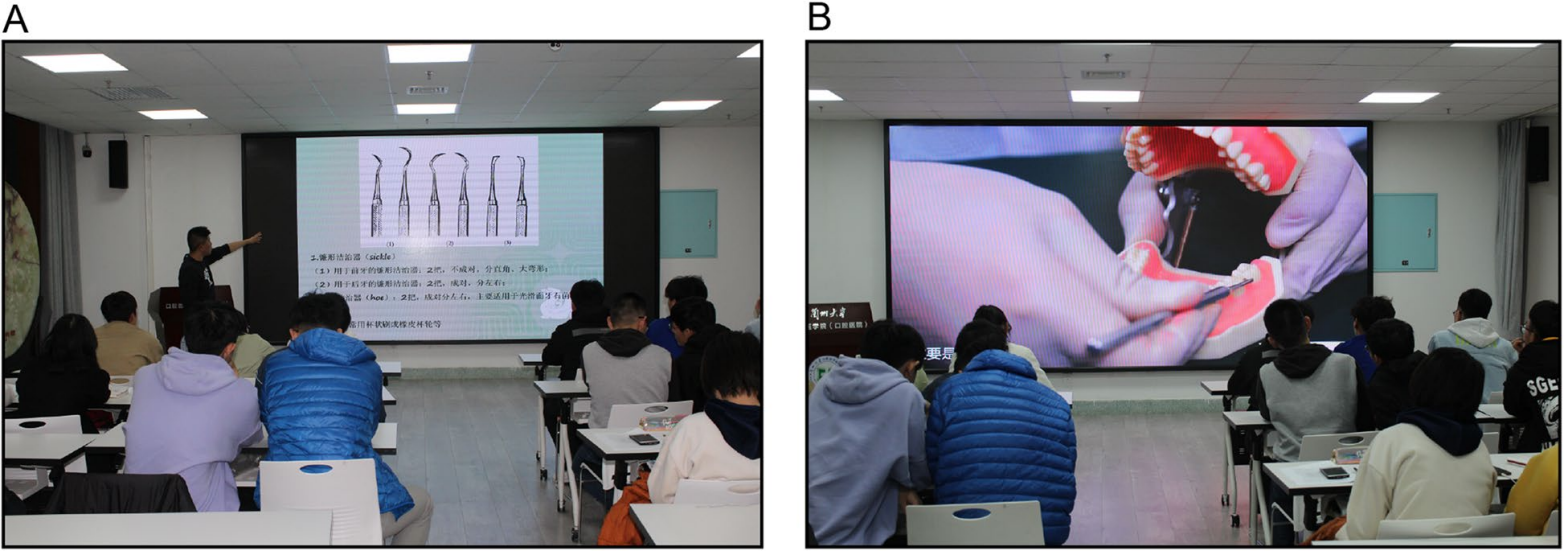
Representative images showing theoretical teaching. **A** Participants were taught theoretical skills for 2 h by clinical dentists with more than 5 years of experience. **B** Participants watch operation training video

A flow chart that illustrates the study design is presented in Fig. 1. Participants attended a 2-h theoretical knowledge classroom session, prior to Exam 1. The session was taught by a senior periodontist with more than 5 years of clinical experience, and participants watched the standardized operation of the teaching video (Chinese Oral Practitioner Practice Skills). The lecture content comprised medical history enquiry, tissue anatomy, aseptic concept, preoperative preparation, instrument selection, comprehensive periodontal examination (such as probing pocket depth (PPD), bleeding on probing (BOP), and clinical attachment level (CAL)), and subgingival scaling (Fig. 2). The lecture was based on the criteria outlined in the Periodontology textbook (4th edition) [23]. The first assessment was conducted to determine the potential learning ability and comprised multiple-choice questions.

#### Operation training

**Fig. 3:**
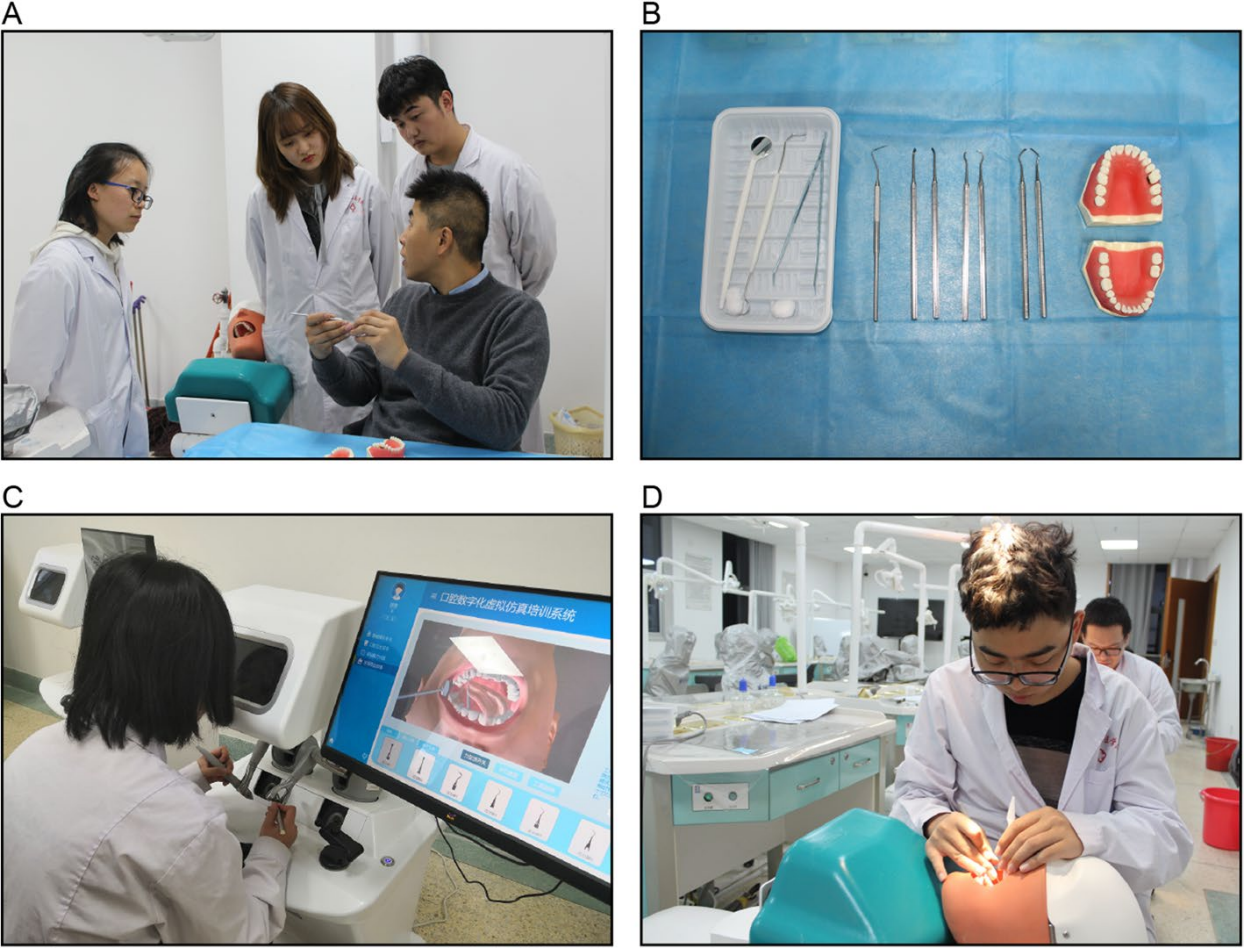
Operation training of supragingival scaling. **A** Key points of operation on the jaw simulator. **B** Manual supragingival scaling tools. **C** Training using the virtual reality system. **D** Training using the jaw simulator

Participants received hands-on training for performing operation that included the following techniques: plaque index record, medical history inquiry, aseptic concept, equipment preparation, chair position adjustment, comprehensive periodontal examination, supragingival scaling, postoperative examination, and oral health education (Fig. 3A/B). The training lasted approximately 8 h in total (2 h/day). The left maxillary central incisor (#21) and right mandibular first molar (#46) were set up as uniform sites. In addition, the training order of groups V-J and J-V were reversed to eliminate the order factor. Furthermore, students in V-J and J-V groups underwent training on the jaw model (NISSIN Dental Products Inc) (Fig. 3B/D) and the VR system (UniDental) (Fig. 3C) to explore whether the order of the two methods affected the training outcomes. The jaw model and VR system training each lasted for 4 h.

#### Operation examination

Participants sat for the second theoretical knowledge exam (that is Exam 2, with the same difficulty as exam) after completing the operation training. The operation process assessment comprised content from the Chinese Oral Physician Licensing Exam, such as supragingival scaling. The assessment was double-blinded and evaluation was performed by 3 professional dentists with 5 years of clinical experience. Items and scoring standards used are presented in Table 1 including preoperative preparation, operation posture, fulcrum, periodontal probe, and supragingival scaling.

**Table 1:** Scaling operation score sheet

Scoring items	Score
***Preparation***	
**Preoperative preparation**	
Dress neatly, asepsis, necessary preoperative instructions	3
Choosing right instruments including periodontal probe and scaler	7
**Operation posture**	
Sit up straightly and stablely	2
The patient's jaw plane is located at or below the elbow	2
Adjust the position according to different teeth position	2
Use of oroscope in the exploration of lingual and palatal side	2
Adjustment of lights in different positions	2
***Intraoperative operation***	
**Holding**	
Improved writing style	5
Combined fulcrum	5
**Fulcrum**	
Alternate use of intraoral and extraoral fulcrum	2
No slippage of instrument	2
Fulcrum moves with the change of teeth position	2
**Periodontal probing**	
The angle of proximal surface probing	5
The angle of lip and palate probing	5
The way of buccal probing	5
The order of probing	5
Correct record	6
**Supragingival scaling**	
Probing and recording of subgingival calculus	6
80 ° angle between blade and tooth surface	5
Wrist force	5
Direction of force	5
Remove the calculus in one piece	6
Continuity of scaling	5
Probe inspection after scaling	6
***Total***	100

#### Supragingival scaling effect

Supragingival scaling is a procedure for removal of supragingival calculus, plaque, and color stains using various instruments. Intraoral examination showed no calculus in the mouths of participants, thus the plaque index was used to explore whether subjects required supragingival scaling. All 60 volunteers underwent periodontal scaling process randomly through pairwise correlation. The supragingival scaling score was then expressed as a percentage using the following equation:

$$\frac{\mathrm{Total}\ \mathrm{plaque}\ \mathrm{index}\ \mathrm{before}\ \mathrm{scaling}-\mathrm{total}\ \mathrm{plaque}\ \mathrm{index}\ \mathrm{after}\ \mathrm{scaling}}{\mathrm{Total}\ \mathrm{plaque}\ \mathrm{index}\ \mathrm{before}\ \mathrm{scaling}}\times 100$$

Turesky modification of the Quigley–Hein index [24], was used to assess the supragingival plaque on six sites (including mesial buccal, median buccal, distal buccal, mesial lingual, median lingual, distal lingual) of each tooth [25] (Fig. 5C/D/E/F). The plaque index uses Quigley-Hein’s improved Turesky plaque index to evaluate plaque on the gums at #21 and #46 levels before and after supragingival scaling procedure. The scores are as follows: 0: no plaque, 1: presence of scattered plaques at the edge of the tooth cervix, 2: continuous thin plaque band visible at the edge of the tooth cervix, not more than 1 mm wide, 3: tooth and neck plaque with a bandwidth greater than 1 mm, but less than 1/3 of the tooth surface, 4: Plaque covering 1/3 to 2/3 of the tooth surface, 5: plaque covering more than 2/3 of the tooth surface (Fig. 5E/F).

#### Questionnaire survey

The degree of satisfaction of participants was determined through a questionnaire using a Likert scale after the teaching session as shown in Table 2. Each item was rated, with a score of 5 indicating “strongly agree,” 4 indicating “agree,” 3 indicating “neither agree nor disagree,” 2 indicating “disagree,” and 1 indicating “strongly disagree”.

**Table 2:** Results of the survey

Project Evaluation Score	Groups (Mean±SD)			
	J	V	J-V	V-J
Course focus	3.53±0.62	3.60±0.71	4.13±0.34^*^	4.07±0.44^*^
Course interest	3.40±0.71	3.60±0.49	4.67±0.47^*^	4.40±0.61^*^
Course richness	3.80±0.40	3.73±0.44	4.33±0.47^*^	4.80±0.40^*^
Combine theory with practice	3.93±0.44	3.73±0.57	4.80±0.40^*^	4.87±0.34^*^
Acquisition of konwledge	3.13±0.72	3.73±0.57	4.20±0.40^*^	4.20±0.54^*^
Improvement of clinical skills	3.87±0.34	3.00±0.89	4.60±0.61^*^	4.47±0.81^*^
The activity of the class atmosphere	3.47±0.72	3.93±0.25	4.33±0.47^*^	4.47±0.62^*^
Improvement of learning motivation	3.27±0.57	3.40±0.49	4.53±0.50^*^	4.33±0.60^*^
Satisfaction with the use of laboratory	3.40±0.80	3.67±0.47	4.47±0.50^*^	4.40±0.49
Interaction between teachers and students	3.67±0.47	3.87±0.34	4.60±0.49^*^	4.67±0.47^*^

* *P*< 0.05 *vs* J groups, one-way ANOVA

### Statistical analysis

Data analysis was performed using SPSS 20 statistical software (IBM Inc., Chicago, IL). Data were presented as mean ± standard deviation (SD). Comparison among the four groups was performed through one-way ANOVA for data that were normally distributed and showed homogeneous variance. Hierarchical data was analyzed using non-parametric Mann-Whitney U Rank Sum test. *P* value less than 0.05 was considered statistically significant.

## Results

### Theoretical knowledge

**Fig. 4:**
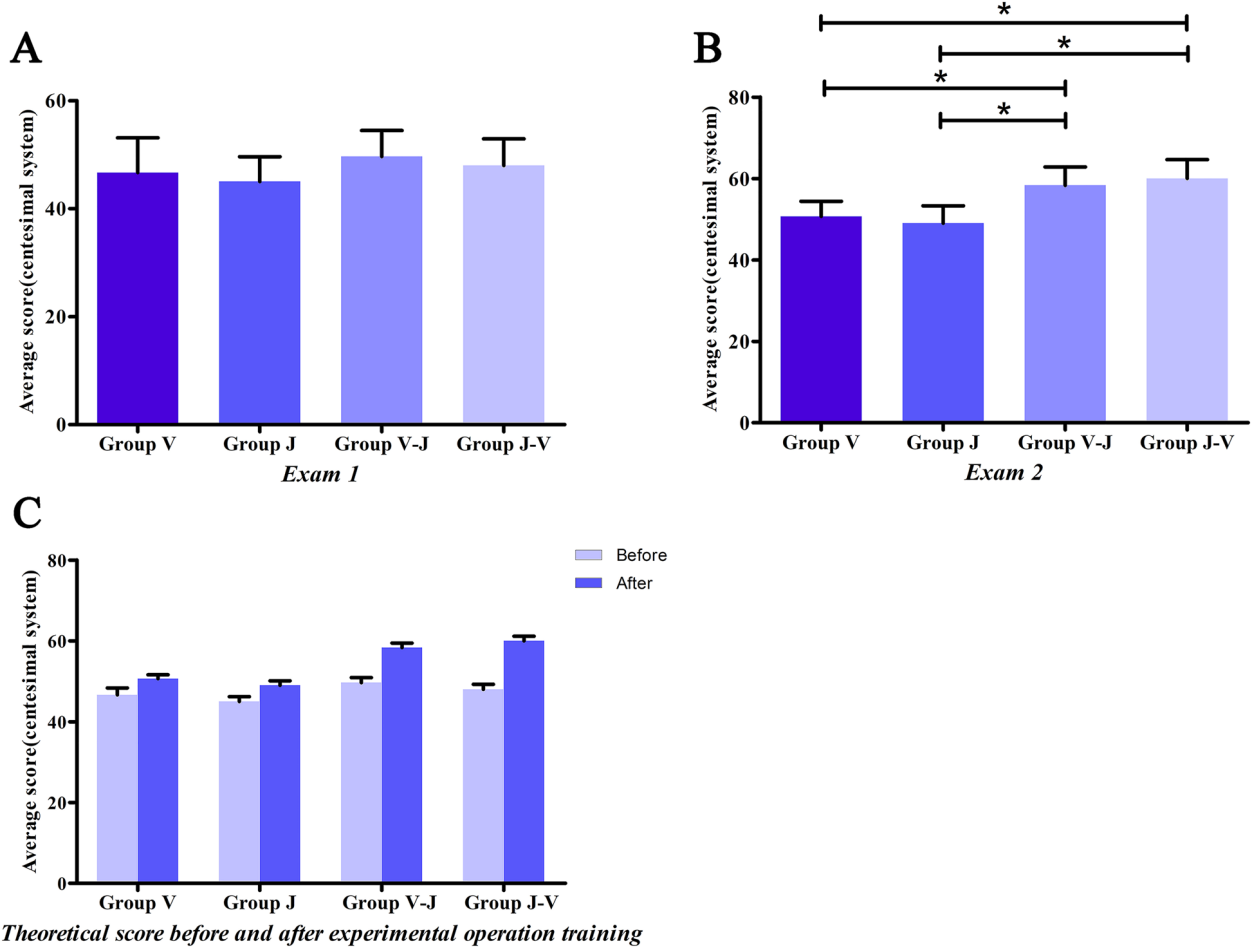
Theoretical scores for different groups in the study. **A** Scores of the first theoretical test showing no significant difference among groups (t-test, one-way ANOVA, correlation analysis and NSK, *P* > 0.05). **B** Scores of Group V-J and J-V which were higher compared with the scores of V and J in the second theoretical test (*P* < 0.05). **C** Comparison of the first and second theoretical scores (*P* < 0.05). Scores from Group V-J were significantly different compared with those of group J-V (*P* < 0.01)

The average score of the first theoretical examination was 47.33 ± 5.40. The result showed no significant differences in the first theoretical examination scores between the four groups (V: 46.67 ± 6.45; J: 45.00 ± 4.63; V-J: 49.67 ± 4.81; J-V: 48.00 ± 4.93) (V vs J, *P* = 0.389; V vs V-J *P* = 0.124; V vs J-V, *P* = 0.49; J vs V-J, *P* = 0.018; J vs J-V, *P* = 0.124; V-J vs J-V, *P* = 0.389; *P* > 0.05, Fig. 4A).

The scores of the second theoretical examination were significantly higher compared with the first theoretical examination scores in each group (V: 50.67 ± 3.72; J: 49.00 ± 4.31; V-J: 60.00 ± 4.47; J-V: 58.33 ± 4.35) (V vs J, *P* = 0.293; V vs V-J *P* = 0.000; V vs J-V, *P* = 0.000; J vs V-J, *P* = 0.000; J vs J-V, *P* = 0.000; V-J vs J-V, *P* = 0.293, Fig. 4B/C). In addition, the individual scores of students in groups V-J and J-V were significantly higher compared with the scores of students in the other two groups (*P* < 0.05, Fig. 4B/C). However, the findings showed no significant differences in academic performance between students in Group V-J and Group J-V.

### Operation assessment

**Fig. 5:**
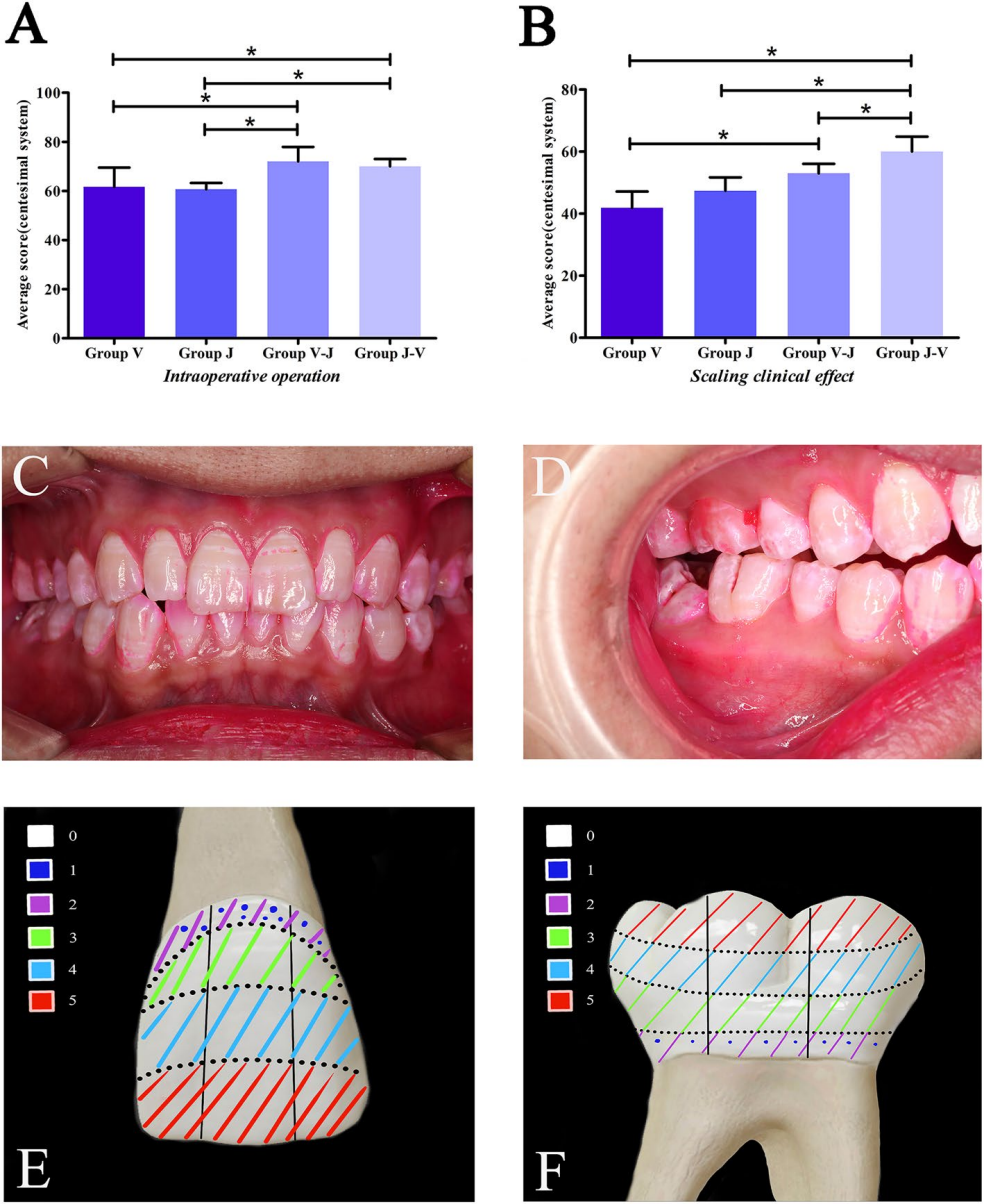
Scores on operational assessment. **A** Group V-J and J-V exhibited lower subjective score compared with the score for V-J and J-V groups (*P* < 0.05). **B** Group J-V showed the best performance on the scaling effect compared with group V (*P* < 0.01) and J (*P* < 0.01). **C/D** Scaling as indicated by periodontal plaque indicator. **E/F** Corresponding scoring standard using Quigley-Hein index

The scoring key points of the operation procedure (Table. 1) were judged by 3 professional clinical doctors. Group V-J (72.00 ± 5.92) and Group J-V (70.00 ± 3.05) showed a relatively better performance on the operation process compared with the other two groups (V: 61.67 ± 7.85; J: 60.67 ± 2.58; *P* < 0.05, Fig. 5A) (V vs J, *P* = 0.608; V vs V-J *P* = 0.000; V vs J-V, *P* = 0.000; J vs V-J, *P* = 0.000; J vs J-V, *P* = 0.000; V-J vs J-V, *P* = 0.306, Fig. 4B/C). The supragingival scaling effect was significantly higher in Group V-J and Group J-V (V-J: 53.00 ± 3.05; J-V: 63.40 ± 4.39) compared with the supragingival scaling effect in Group J and Group V (V: 41.90 ± 5.23; J: 47.40 ± 4.31; *P* < 0.01). Notably, Group J-V indicated the highest efficacy (Fig. 5B). (V vs J, *P* = 0.121; V vs V-J, *P* = 0.002; V vs J-V, *P* = 0.000; J vs V-J, *P* = 0.114; J vs J-V, *P* = 0.001; V-J vs J-V, *P* = 0.035, Fig. 4B/C).

### Results from the questionnaire

The scores on the Student’s Satisfaction Questionnaire showed that participants in Group V-J and Group J-V were highly satisfied with the training compared with subjects in the other groups (Table 2). The item with the lowest score in Group J was “Acquisition of knowledge”, and the findings showed that only one-third of the students were satisfied. The item with the lowest score in Group V was “Improvement of clinical skills”, with 27% of the students reporting dissatisfaction, and 40% of the participants who were dissatisfied chose “neither agree nor disagree”. The highest-rated item in Group V-J and J-V groups was “Combine theory with practice”. The findings indicated that the training method using the jaw model combined with virtual simulation system had excellent results.

## Discussion

Periodontal disease can cause gingival atrophy, alveolar bone absorption, tooth mobility and loss, even affect the development of systemic diseases. Treatment of periodontal disease is important to alleviate advances into other conditions. Notably, periodontal disease is mainly treated using standard treatment operation. Therefore, it is important to conduct preclinical operation training for stomatological students. The primary objective of the current study was to compare the effect of virtual simulation and jaw model on development of preclinical periodontal skills in undergraduate students. In addition, the study sought to explore effectiveness of virtual simulation in periodontal preclinical teaching unit, to identify effective approaches and circumvent the shortcomings of traditional teaching methods thus allowing students to easily practice new skills [26].

The findings of the current study indicated that combination of virtual simulation and jaw model was superior compared with traditional teaching methods, either after promoting theoretical knowledge or acquisition of periodontal supragingival scaling of clinical skills. In addition, the findings showed that the order of the methods (J-V vs V-J) affected effectiveness of clinical teaching, with significant effect observed for the supragingival scaling procedure owing to students’ abilities to purposefully and selectively master skills in virtual stimulation systems after jaw model training. Transition from theoretical medicine into clinical practice is challenging, thus there is an urgent need for simulation training methods that integrate theoretical skills and practical skills [27]. A previous study explored role of communication between physician and patient by simulating different clinical specialties which significantly boosted their memory of theoretical skills [28]. Murbay [29] assessed undergraduate performance by introducing a randomized setting using a Moog Simodont virtual system within the preclinical stage and reported that it significantly improved student’s operation level. This type of training is valuable for students in training programs and for undergraduate training, to improve mastery of tooth preparation skills using virtual simulation [30]. In addition, de Boer [4] found that after sufficient amount of time preclinical training by virtual force feedback, the student became more confident and acquired a manual dexterity skill, thus realizing the transition between preclinical training and clinical practice. The experimental and modeling results of the current study indicate that virtual simulation is important in improving operation ability. Notably, a single virtual simulation training cannot achieve significant effects owing to differences in the system used, proficiency of the system, and the particularity of dentistry [31]. Previous studies report that virtual technology offers multiple advantages in education, including improved efficiency and quality of study through feedback signals to the brain, sufficient and free training time, and accurate and automatic training data [32]. However, virtual technology should not be used as an alternative to traditional methods due to features such as excessive critical feedback, lack of personal contact, and technical hardware difficulties that are associated with VR-based training, or lack of concrete experience in the training systems [33]. Plessas [34] reported that guidance and evaluation by professional teachers are indispensable and virtual systems cannot fully replace traditional training courses. Al-Saud et al. [35] randomly assigned 63 people without oral professional training into 3 groups as follows: device feedback group, instructor feedback group, and instructor device feedback group. The findings showed that skills and error rate in the instructor device feedback group were significantly improved compared with those in the other two groups.

Combination of professional guidance and feedback from a virtual simulation system (such as VR) significantly improves learning and mastery of basic oral operations in junior students, which is consistent with the findings of the current study. Notably, the optimal sequence for training with jaw model and virtual simulation has not been elucidated. In the current study, the best supragingival scaling effect was observed in Group J-V. Situational training of virtual simulation can achieve good tactile feedback on the cleaning force, implying that the jaw model can achieve good cleaning feeling and force control, resulting in good reproduction of supragingival scaling effect. Moreover, the virtual simulation system effectively interacts with students and simulates clinical diagnosis and treatment process. The jaw model is a physical model that can be operated easily and intuitively, however, it lacks clinical situational representation, which is important for the training of clinical thinking. Notably, VR is characterized by clinical situational representation making it more effective compared with the jaw model. Neurophysiological studies report that this discrepancy may be associated with differences in personal psychomotor skills [36].

The strengths and limitations of the current study need consideration. The methodology and findings address the shortcomings of VR periodontal education reported in previous literature and provide a useful reference for further development of medical teaching models. The present study used the Turesky modification of the Quigley–Hein plaque index gathering student’s training outcomes instead of using direct practical clinical operation of patients. This method is used to explore the teaching effect of supragingival scaling. Nevertheless, our study also had some limitations. Firstly, the therapeutic effects of subgingival scaling could not be monitored over a short period of time. Also, the study only enrolled a few undergraduate students. The approach adopted in this study was inevitably one-sided compared with the general strategies currently used to explore periodontal treatment effects, such as probing depth and attachment loss. And the duration of training of the experimental subjects was not sufficient to fully predict long-term application effect of the different teaching methods. Further studies should be conducted to explore the long-term effects of the virtual stimulation system, including the optimal application period for the teaching process. Moreover, different manipulation systems of VR may result in bias [37], and more realistic virtual simulation equipment is required to develop.

## Conclusion

The findings of the current study indicate that a combination of VR and jaw model during periodontal preclinical training can increase students’ grades and significantly improve their professional skills. In addition, the jaw model should be applied prior to VR to optimize learning skills in basic periodontal education. The present study provides a basis to improve future periodontal preclinical training strategies.

## Supplementary Information

**Additional file 1.**

## Data Availability

The datasets used and/or analyzed during the current study are available from the corresponding author on reasonable request.

## Abbreviations

VR: Virtual reality
PPD: Probing pocket depth
BOP: Bleeding on probing
CAL: Clinical attachment level
