# Supplementary material for: Effectiveness of virtual simulation and jaw model for undergraduate periodontal teaching
Source: BMC Med Educ. 2021 Dec 14;21:616. doi: 10.1186/s12909-021-03064-1 (PMC8672555; doi:10.1186/s12909-021-03064-1)
Supplement: Supplementary file 1 — Additional file 1. [file 12909_2021_3064_MOESM1_ESM.docx]

**Appendix:**

**Table S1.** Results of the survey in Group J

| **Project Evaluation Score** | **Score (Percentage)** | | | | |
| --- | --- | --- | --- | --- | --- |
|  | 5 | 4 | 3 | 2 | 1 |
| Course focus | N/A | 47% | 47% | 6% | N/A |
| Course interest | N/A | 53% | 33% | 14% | N/A |
| Course richness | N/A | 80% | 20% | N/A | N/A |
| Combine theory with practice | N/A | 93% | 7% | N/A | N/A |
| Acquisition of konwledge | N/A | 27% | 60% | 13% | N/A |
| Improvement of clinical skills | N/A | 87% | 13% | N/A | N/A |
| The activity of the class atmosphere | N/A | 50% | 43% | 7% | N/A |
| Improvement of learning motivation | N/A | 27% | 73% | N/A | N/A |
| Satisfaction with the use of laboratory | 33% | 40% | 14% | 13% | N/A |
| Interaction between teachers and students | N/A | 67% | 33% | N/A | N/A |

Note: 5, strongly agree; 4, agree; 3, neither agree nor disagree; 2, disagree; 1, strongly disagree; N/A, null.

**Table S2.** Results of the survey in Group V

| **Project Evaluation Score** | **Score (Percentage)** | | | | |
| --- | --- | --- | --- | --- | --- |
|  | 5 | 4 | 3 | 2 | 1 |
| Course focus | 6% | 47% | 47% | N/A | N/A |
| Course interest | N/A | 67% | 33% | N/A | N/A |
| Course richness | N/A | 73% | 27% | N/A | N/A |
| Combine theory with practice | 6% | 60% | 34% | N/A | N/A |
| Acquisition of konwledge | N/A | 81% | 13% | 6% | N/A |
| Improvement of clinical skills | N/A | 33% | 40% | 27% | N/A |
| The activity of the class atmosphere | N/A | 94% | 6% | N/A | N/A |
| Improvement of learning motivation | N/A | 40% | 60% | N/A | N/A |
| Satisfaction with the use of laboratory | N/A | 67% | 33% | N/A | N/A |
| Interaction between teachers and students | N/A | 87% | 13% | N/A | N/A |

Note: 5, strongly agree; 4, agree; 3, neither agree nor disagree; 2, disagree; 1, strongly disagree; N/A, null.

**Table S3.** Results of the survey in Group J-V

| **Project Evaluation Score** | **Score (Percentage)** | | | | |
| --- | --- | --- | --- | --- | --- |
|  | 5 | 4 | 3 | 2 | 1 |
| Course focus | 13% | 87% | N/A | N/A | N/A |
| Course interest | 67% | 33% | N/A | N/A | N/A |
| Course richness | 33% | 67% | N/A | N/A | N/A |
| Combine theory with practice | 80% | 20% | N/A | N/A | N/A |
| Acquisition of konwledge | 20% | 80% | N/A | N/A | N/A |
| Improvement of clinical skills | 60% | 40% | N/A | N/A | N/A |
| The activity of the class atmosphere | 33% | 67% | N/A | N/A | N/A |
| Improvement of learning motivation | 53% | 47% | N/A | N/A | N/A |
| Satisfaction with the use of laboratory | 47% | 53% | N/A | N/A | N/A |
| Interaction between teachers and students | 67% | 33% | N/A | N/A | N/A |

Note: 5, strongly agree; 4, agree; 3, neither agree nor disagree; 2, disagree; 1, strongly disagree;

N/A, null.

**Table S4.** Results of the survey in Group V-J

| **Project Evaluation Score** | **Score (Percentage)** | | | | |
| --- | --- | --- | --- | --- | --- |
|  | 5 | 4 | 3 | 2 | 1 |
| Course focus | 14% | 80% | 6% | N/A | N/A |
| Course interest | 47% | 47% | 6% | N/A | N/A |
| Course richness | 80% | 20% | N/A | N/A | N/A |
| Combine theory with practice | 87% | 13% | N/A | N/A | N/A |
| Acquisition of konwledge | 20% | 80% | N/A | N/A | N/A |
| Improvement of clinical skills | 40% | 60% | N/A | N/A | N/A |
| The activity of the class atmosphere | 47% | 53% | N/A | N/A | N/A |
| Improvement of learning motivation | 40% | 60% | N/A | N/A | N/A |
| Satisfaction with the use of laboratory | 40% | 60% | N/A | N/A | N/A |
| Interaction between teachers and students | 67% | 33% | N/A | N/A | N/A |

Note: 5, strongly agree; 4, agree; 3, neither agree nor disagree; 2, disagree; 1, strongly disagree; N/A, null.
